# Effect of rapid antiretroviral therapy initiation on loss to follow-up, mortality, and virologic failure among people with human immunodeficiency virus under the treat-all policy in China: analysis of routine data

**DOI:** 10.3389/fcimb.2025.1736328

**Published:** 2026-01-13

**Authors:** Juan Jin, Songnan Pan, Xinyan Jing, Huanhuan Ba, Yuan Zhang, Jiajia Li, Jinling Yin, Peipei Luo, Haohua Hou, Kangxiao Ma

**Affiliations:** 1 Department of Infectious Diseases, Xi’an Eighth’s Hospital, Xi’an, Shaanxi, China; 2 Department of Infectious Diseases, Infectious Disease Hospital of Heilongjiang Province, Harbin, Heilongjiang, China

**Keywords:** antiretroviral therapy, China, HIV, loss to follow-up, mortality, rapid initiation, virologic failure

## Abstract

**Objectives:**

Since 2016, China has provided timely HIV antiretroviral therapy (ART) under the treat-all policy. This study aimed to evaluate the impact of rapid ART initiation (≤7 days post-HIV diagnosis) on loss to follow-up (LTFU), mortality, and virologic failure compared with that of delayed ART.

**Methods:**

This study included adults with ART-naive HIV infection in Xi’an, China, between 2016 and 2022. Kaplan–Meier analysis was used to examine LTFU and death time for rapid and delayed ART initiation. Moreover, multivariate Cox regression was employed to evaluate the correlation between rapid ART initiation and LTFU/mortality, while logistic regression was utilized to assess the association between rapid ART and 12-month virologic failure.

**Results:**

Of the 6992 participants, 770 (11.0%) initiated ART ≤7 days postdiagnosis. The percent of ART initiations in the first week postdiagnosis quadrupled from 4.2% in 2016 to 19.7% in 2022. The LTFU rate for rapid ART initiators was comparable to that in the 8–29- (*P* = 0.132) and ≥30-day groups (*P* = 0.432). Mortality was notably decreased in the rapid ART group (0.0%) than in the 8–29- (1.5%) and ≥30-day groups (2.2%). The rapid ART initiators demonstrated lower odds of developing virologic failure compared with delayed ART initiators (aOR: 0.50; 95% CI: 0.26–0.89; *P* = 0.028; ≤7 days versus ≥30 days).

**Conclusions:**

Under China’s treat-all policy, rapid ART initiation showed equivalent LTFU but lower mortality and virologic failure. Chinese HIV patients may benefit from rapidly ART, but they require more intensive, tailored counseling to remain in treatment.

## Introduction

Over the past 20 years, antiretroviral therapy (ART) has proven a critical component in fighting against the human immunodeficiency virus (HIV) pandemic, which causes the acquired immunodeficiency syndrome (AIDS). Increased usage of ART has significantly declined AIDS-associated mortality and morbidity, in addition to reduced HIV transmission (Hou et al., 2022; Weiser et al., 2022; Broyles et al., 2023). China has experienced a dramatic growth in the number of people with HIV (PWH), who receive free government-sponsored ART since the National Free Antiretroviral Treatment Program (NFATP) was launched in 2003 (Cao et al., 2020). By the end of 2020, 92.9% of the 1.05 million PWH in China were receiving ART (He, 2021). The World Health Organization (WHO) recommended a “treat-all” (universal test and treat) policy for all PWH in 2015, enabling all PWH to receive ART irrespective of their CD4 count (Labhardt et al., 2023). Subsequent clinical trial studies on same-day and rapid ART resulted in new WHO guidelines in 2017, recommending that PWH without signs of opportunistic infections should begin ART ≤7 days or on the same day post-HIV diagnosis (Amanyire et al., 2016; Rosen et al., 2016; Committee, W.G.R, 2017).

In 2016, China implemented a treat-all policy, recommending early initiation of ART upon diagnosis voluntarily (Cao et al., 2020), and launched several programs to accelerate ART initiation, improve care retention via enlarged diagnostics and therapies, and enhance the care cascade (Zhao et al., 2019). The Chinese guidelines updated in 2021 recommend the rapid ART initiation or same-day ART (Aids et al., 2021). Although the original WHO recommendation was predominantly derived from randomized trial outcomes, the extensive implementation of rapid ART offers a number of observational studies to evaluate the practical consequences of this strategy. While same-day and rapid ART clearly increase treatment uptake and shorten time to viral suppression, their effects on longer-term engagement in care—particularly retention and virologic failure—remain mixed and are not yet fully understood, especially beyond the first year of treatment (Agroia et al., 2025; Duggan et al., 2025). Extensive cohort studies conducted in China from 2011 to 2015 revealed that immediate ART (≤30 days postdiagnosis) among PWH was associated with a remarkable decrease in overall mortality as well as a marked decrease in treatment dropout and virologic failure (Zhao et al., 2018, 2019). In contrast to the Chinese study results and former randomized trial outcomes, some observational studies—often conducted in different settings or with distinct populations—have reported that same-day ART correlates with poorer rates of virologic suppression or post-ART engagement in care. These findings may reflect contextual challenges such as health system readiness, patient preparedness, or follow-up support rather than the inherent value of rapid ART (Joseph Davey et al., 2020; Kerschberger et al., 2021; Ross et al., 2023). After implementing the national “treat-all” policies, most studies on rapid ART involve randomized trials and observational studies conducted in Africa (Labhardt et al., 2023). Reaching regional epidemic control targets requires a better understanding of whether rapid ART affects clinical outcomes under the treat-all policy in China. Although prior studies in China have examined rapid ART in controlled or shorter-term settings, there remains a gap in understanding its real-world, longer-term impact on loss to follow-up (LTFU), mortality, and virologic failure under the treat-all policy in a large urban cohort over a multi-year period (Wu et al., 2015; Wang et al., 2024; Xia et al., 2024). Such real-world evidence is required to translate clinical trial results into complicated medical systems and guide the development of interventions to assist policy implementation procedures. Therefore, we investigated the association between rapid ART initiation and LTFU, mortality, and virologic failure in PWH recruited from a large real-world HIV/AIDS cohort in Xi’an, China.

## Methods

### Study population and data collection

This retrospective observational study included newly diagnosed HIV-positive adults with records of their first ART initiated between June 2016 (when China adopted the treat-all policy for all PWH) and December 2022 in Xi’an, a megacity in China with a moderate prevalence of HIV (PWH > 10000). The Human Medical Ethics Committee of Xi’an Eighth Hospital approved this study as conducted under the Declaration of Helsinki. Each patient provided informed consent during diagnosis to allow clinical research using their clinical records. Exclusion criteria included (1) diagnosis before the nationwide implementation of universal HIV treatment, (2) <18 years old, (3) ART experience, (4) <12 months between enrollment and the latest visit, (5) never initiated ART, and (6) transferred out. Data were collected from the Chinese Centre for Disease Control and Prevention (CDC)’s HIV/AIDS Comprehensive Response Information Management System (Mao et al., 2010) and electronic medical records of the hospital. We obtained data on sex, age, marital status, HIV diagnosis dates, transmission routes, entry to HIV care, viral load, CD4 count, ART prescription date, visit, transfer to other cities, and death. Participants were categorized based on the date of ART initiation following HIV diagnosis: ≤7, 8–29, and ≥30 days.

### Outcome measures

Rapid ART initiation was defined as ART initiation ≤7 days postdiagnosis, consistent with WHO guidelines and operational research definitions (WHO, 2017). The primary outcome was LTFU at 12 months after enrollment, defined as having no documented interaction with healthcare facilities for >90 consecutive days before the 12-month mark, among those who had not died or been transferred out (Onoya et al., 2021; Kimanga et al., 2022). The secondary outcome was all-cause mortality. Additionally, we evaluated 12-month virologic failure (defined as viral load ≥200 copies/mL at the first annual test post-ART initiation) as a tertiary outcome.

### Statistical analysis

All analyses were performed using version 26.0 of the Statistical Package for the Social Sciences (SPSS, Chicago, IL). Kruskal–Wallis tests were utilized to examine differences in medians, whereas chi-square tests were utilized to evaluate differences in categorical variables. For time-to-event studies, the follow-up time was assessed in days from the start of ART to the final follow-up. Furthermore, the Kaplan–Meier method was performed to determine the LTFU/death time and compare the timing of ART initiation. We assessed the risk of LTFU and death ≤12 months after HIV diagnosis using Cox regression models. The hazard ratio (HR) and adjusted HR (aHR) were reported with 95% confidence intervals (CI). We estimated the risks of virologic failure using multivariate logistic regression models, yielding odds ratio (OR), adjusted OR (aOR), and 95% CI. All *P*-values are two-sided, where *P*-values of <0.05 indicate statistical significance.

## Results

### Participant characteristics

**Figure 1:**
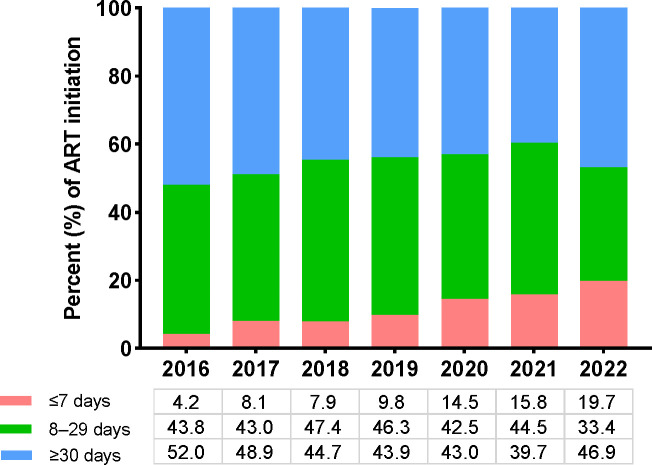
Trends in ART initiation after HIV diagnosis from June 2016 to December 2022. *ART* antiretroviral therapy, *HIV* human immunodeficiency virus.

This study included 6992 participants (**Table 1**). Of these, 770 (11.0%), 3031 (43.4%), and 3191 (45.6%) have initiated ART in ≤7, 8–29, and ≥30 days postdiagnosis. The median times between being diagnosed with HIV and ART initiation for the three groups were 6 (interquartile range [IQR]: 5–7), 19 (IQR: 14–23), and 71 days (IQR: 42–238, data not shown), respectively. The rapid ART initiators were predominantly male (92.9% rapid ART) and aged 30–49 years (53.0% rapid ART). Additionally, 24.2% of individuals aged ≥50 years initiated ART ≤7 days postdiagnosis, followed by those in the 18–29 age group. During enrollment, the median baseline CD4 count was similar among rapid ART (289 cells/μL, IQR: 139–413) and 8–29-day groups (IQR: 158–422) but lower than the ≥30-day group (311 cells/μL, IQR: 156–460). Moreover, 16.2% of the rapid initiators’ baseline CD4 count were ≥500 cells/μL, similar to the 8–29-day group (16.0%) but lower than the ≥30-day group (19.4%). Approximately two-thirds (69.9%) of the rapid ART initiators acquired HIV through homosexual contact, and nearly half (53.0%) were single. People who quickly initiated ART (32.5%) were more likely to select an INSTI-based regimen than those who did at 8–29 (17.6%) or ≥30 (19.1%) days. The percentage of ART initiations during the first week postdiagnosis increased significantly, more than quadrupling from 4.2% to 19.7% of all initiations, between 2016 and 2022 (**Figure 1**).

**Table 1:** Participant characteristics.

Variables	Total (n = 6992)	≤7 days ART (n = 770)	8–29 days ART (n = 3031)	≥30 days ART (*n* = 3191)	Statistic	*P*
Sex, *n* (%)						
Female	497 (7.1)	56 (7.3)	201 (6.6)	240 (7.5)	1.899	0.387
Male	6495 (92.9)	714 (92.7)	2830 (93.4)	2951 (92.5)		
Age at Enrollment (years), *n* (%)						
Median (IQR)	38 [31, 50]	37 [30, 49]	39 [32, 52]	37 [31, 49]	27.191	<0.001
18-29	1345 (19.3)	176 (22.9)	572 (18.9)	597 (18.7)	31.362	<0.001
30-49	3806 (54.4)	408 (53.0)	1573 (51.9)	1825 (57.2)		
≥50	1841 (26.3)	186 (24.2)	886 (29.2)	769 (24.1)		
Enrollment CD4, (cells/μL), *n* (%)						
Median (IQR)	297 [156, 439]	289 [139, 413]	288 [158, 422]	311 [156, 460]	16.575	<0.001
<200	2216 (31.7)	258 (33.5)	969 (32.0)	989 (31.0)	14.536	0.006
200–499	3546 (50.7)	387 (50.3)	1577 (52.0)	1582 (49.6)		
≥500	1230 (17.6)	125 (16.2)	485 (16.0)	620 (19.4)		
Transmission route, *n* (%)						
Homosexual	4714 (67.4)	538 (69.9)	2092 (69.0)	2084 (65.3)	50.563	<0.001
Heterosexual	2049 (29.3)	217 (28.2)	857 (28.3)	975 (30.6)		
Injection drug use	63 (0.9)	3 (0.4)	6 (0.2)	54 (1.7)		
Unknown	166 (2.4)	12 (1.6)	76 (2.5)	78 (2.4)		
Marital status, *n* (%)						
Single	3566 (51.0)	408 (53.0)	1443 (47.6)	1715 (53.7)	31.240	<0.001
Married/cohabiting	2412 (34.5)	239 (31.0)	1147 (37.8)	1026 (32.2)		
Divorced/widowed	1014 (14.5)	123 (16.0)	441 (14.5)	450 (14.1)		
Initial ART regimen						
NNRTI-based	5438 (77.8)	505 (65.6)	2439 (80.5)	2494 (78.2)	92.317	<0.001
PI-based	162 (2.3)	15 (1.9)	60 (2.0)	87 (2.7)		
INSTI-based	1392 (19.9)	250 (32.5)	532 (17.6)	610 (19.1)		

*ART*, antiretroviral therapy; *IQR*, interquartile ranges; *NNRTI*, non-nucleoside reverse transcriptase inhibitor; *PI*, protease inhibitor; *INSTI*, integrase inhibitor.

### Loss to follow-up

LTFU was detected in 152 (2.2%) PWH 12 months following enrollment, with a median duration to LTFU of 122 days (IQR: 69-250). LTFU consisted of 2.3% (*n* = 18) of individuals taking rapid ART following a median duration of 55 days (IQR: 25–277). Delayed initiators demonstrated a longer median duration to LTFU of 164 (IQR: 75–278) and 116 days (IQR: 88–243) for the 8–29-day and ≥30-day groups (data not shown), respectively.

**Figure 2:**
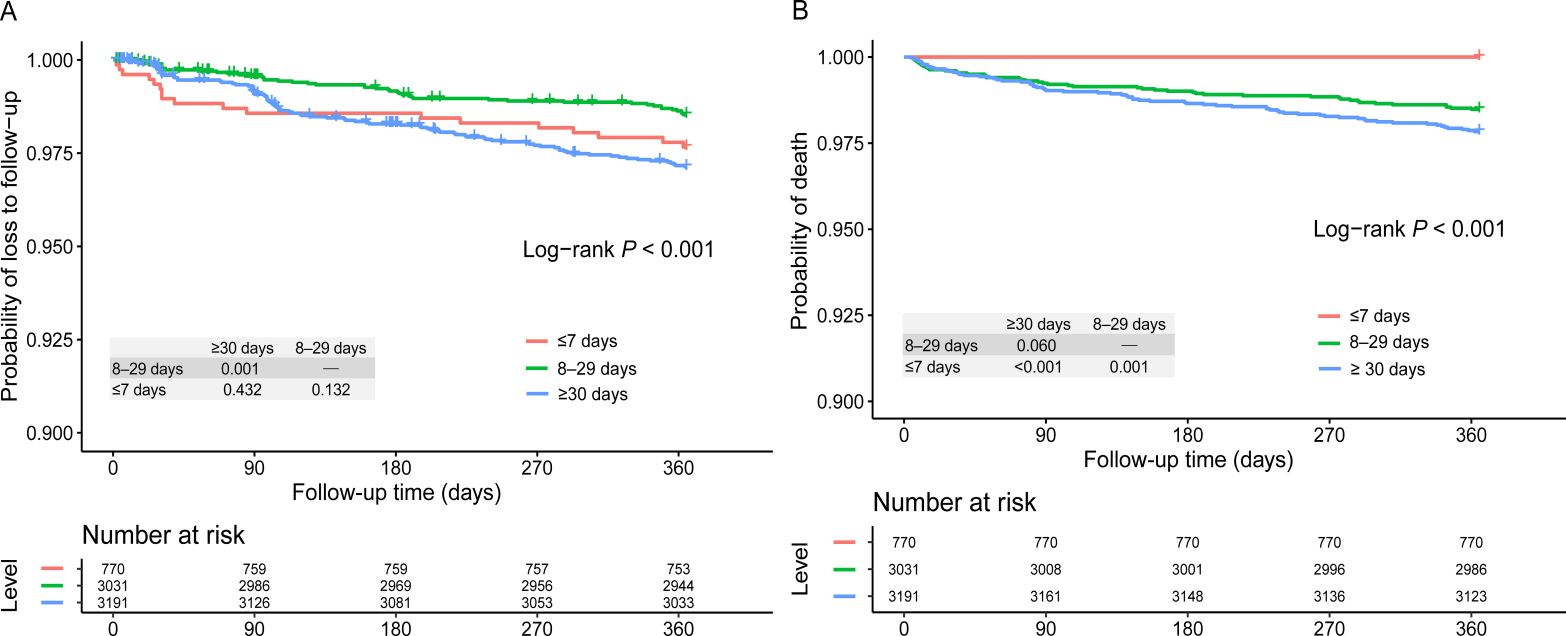
Kaplan–Meier analysis of loss to follow-up **(A)** and mortality **(B)** stratified by ART initiation postdiagnosis timing (≤7, 8–29, and ≥30 days). *ART* antiretroviral therapy.

The Kaplan–Meier analysis revealed comparable LTFU rates for rapid ART initiators to that in the 8–29- (*P* = 0.132) and ≥30-day groups (*P* = 0.432, **Figure 2A**). However, the LTFU rate in the 8–29-day group was considerably reduced compared to the ≥30-day group (*P* = 0.001). The multivariate Cox regression analysis showed that the 8–29-day group had a lower risk of LTFU (aHR: 0.50; 95% CI: 0.34–0.71; *P* < 0.001; 8–29 vs. ≥30 days) controlling for age, sex, enrollment CD4 count, transmission route, marital status, and initial ART regimen (**Table 2**). The hazard of LTFU was greater among people with an unknown transmission route (aHR: 2.40; 95% CI: 1.37–4.18; *P* = 0.002; unknown vs. homosexual), and those ≥50 years (aHR: 2.75; 95% CI: 1.43–5.27; *P* = 0.002) versus those aged 18–29 years.

**Table 2:** Cox regression for loss to follow-up.

Characteristics	Univariate		Multivariate	
	HR (95% CI)	*P*	aHR (95% CI)	*P*
ART initiation group				
≥30 days	1		1	
8–29 days	0.51 (0.35, 0.73)	<0.001	0.50 (0.34, 0.71)	<0.001
≤7 days	0.82 (0.49, 1.35)	0.432	0.85 (0.51, 1.41)	0.518
Sex				
Female	1		1	
Male	0.97 (0.53, 1.8)	0.934	1.38 (0.71, 2.67)	0.344
Age at enrollment (years)				
18–29	1		1	
30–49	1.03 (0.64, 1.66)	0.892	1.09 (0.66, 1.80)	0.728
≥50	2.04 (1.26, 3.29)	0.004	2.75 (1.43, 5.27)	0.002
Enrollment CD4, (cells/μL)				
<200	1		1	
200–499	0.7 (0.49, 0.99)	0.041	0.76 (0.54, 1.09)	0.134
≥500	0.67 (0.41, 1.08)	0.100	0.74 (0.45, 1.21)	0.226
Transmission route				
Homosexual	1		1	
Heterosexual	1.32 (0.93, 1.85)	0.118	1.30 (0.90, 1.90)	0.166
Injection drug use	2.51 (0.79, 7.93)	0.117	1.44 (0.45, 4.62)	0.544
Unknown	2.68 (1.3, 5.51)	0.008	2.40 (1.14, 5.03)	0.021
Marital status				
Single	1		1	
Married/cohabiting	1.2 (0.85, 1.7)	0.302	0.63 (0.39, 1.04)	0.071
Divorced/widowed	1.2 (0.76, 1.91)	0.434	0.66 (0.37, 1.16)	0.148
Initial regimen				
NNRTI-based	1		1	
PI-based	1.45 (0.59, 3.56)	0.412	1.26 (0.51, 3.09)	0.618
INSTI-based	0.92 (0.61, 1.39)	0.687	0.89 (0.58, 1.35)	0.569

*ART*, antiretroviral therapy; *HR*, hazard ratio; *CI*, confidence intervals; *aHR*, adjusted hazard ratio; *IQR*, interquartile ranges; *NNRTI*, non-nucleoside reverse transcriptase inhibitor; *PI*, protease inhibitor; *INSTI*, integrase inhibitor.

### Mortality

Mortality was significantly lower in rapid ART individuals (*n* = 0, 0.0%) compared with those in the 8–29- (*n* = 46, 1.5%; *P* = 0.001) and ≥30-day groups (*n* = 69, 2.2%; *P* < 0.001) (**Figure 2B**). The Cox regression analysis of mortality indicated that the 8–29-day group was protective (aHR: 0.62, 95% CI: 0.43–0.91, 8–29 days vs. ≥30 days) of mortality (**Table 3**). Further, individuals with a higher CD4 count had a decreased risk of death (aHR: 0.15, 95% CI: 0.09–0.24, 200–499 vs. <200, *P* < 0.001; aHR: 0.10, 95% CI: 0.04–0.28, ≥500 vs. <200, *P* < 0.001). The mortality risk was elevated in PWH ≥50 years old (aHR: 4.25, 95% CI: 1.64–10.99, *P* = 0.003; vs. those aged 18–29 years), with an unknown transmission route (aHR: 2.79; 95% CI: 1.39–5.59; *P* = 0.004; unknown vs. homosexual), and those taking a protease inhibitor-containing regimen (aHR: 2.19, 95% CI: 1.05–4.05, *P* = 0.036; vs. those taking a nonnucleoside reverse transcriptase inhibitor[NNRTI]-based regimen), postadjustment.

**Table 3:** Cox regression for death.

Characteristics	Univariate		Multivariate	
	HR (95% CI)	*P*	aHR (95% CI)	*P*
ART initiation group				
≥30 days	1		1	
8–29 days	0.70 (0.48, 1.02)	0.061	0.62 (0.43, 0.91)	0.014
≤7 days	0.00 (0.00, Inf)	0.990	0.00 (0.00, Inf)	0.991
Sex				
Female	1		1	
Male	0.55 (0.32, 0.97)	0.037	0.98 (0.53, 1.81)	0.942
Age at Enrollment (years)				
18–29	1		1	
30–49	1.87 (0.84, 4.2)	0.128	1.27 (0.54, 2.96)	0.584
≥50	7.53 (3.46, 16.37)	<0.001	4.25 (1.64, 10.99)	0.003
Enrollment CD4, (cells/μL)				
<200	1		1	
200–499	0.12 (0.07, 0.2)	<0.001	0.15 (0.09, 0.24)	<0.001
≥500	0.08 (0.03, 0.21)	<0.001	0.10 (0.04, 0.28)	<0.001
Transmission route				
Homosexual	1		1	
Heterosexual	1.83 (1.24, 2.7)	0.002	1.26 (0.82, 1.94)	0.286
Injection drug use	1.28 (0.18, 9.27)	0.804	0.57 (0.08, 4.20)	0.584
Unknown	5.04 (2.58, 9.87)	0.000	2.79 (1.39, 5.59)	0.004
Marital status				
Single	1		1	
Married/cohabiting	3.08 (1.99, 4.76)	<0.001	0.86 (0.47, 1.57)	0.622
Divorced/widowed	2.72 (1.58, 4.68)	<0.001	0.91 (0.47, 1.77)	0.782
Initial regimen				
NNRTI-based	1		1	
PI-based	3.3 (1.6, 6.82)	0.001	2.19 (1.05, 4.55)	0.036
INSTI-based	1.13 (0.72, 1.78)	0.595	1.00 (0.63, 1.58)	0.998

*ART*, antiretroviral therapy; *HR*, hazard ratio; *CI*, confidence intervals; *aHR*, adjusted hazard ratio; *IQR*, interquartile ranges; *NNRTI*, non-nucleoside reverse transcriptase inhibitor; *PI*, protease inhibitor; *INSTI*, integrase inhibitor.

### Virologic failure

After eliminating those who were LTFU or died, 184 were identified to have virologic failure in this cohort of participants. The virologic failure rates were 2.7% overall (184/6725); 1.6% (12/752), 2.5% (75/2941), and 3.2% (97/3032) in the ≤7-, 8–29-, and ≥30-day groups (*P* = 0.054, data not shown), respectively. Logistic regression analysis revealed that rapid ART initiators had lower odds of virologic failure than delayed ART initiators (aOR: 0.50; 95% CI: 0.26–0.89; *P* = 0.028; ≤7 days vs. ≥30 days) (**Table 4**). Lower odds of virologic failure were also seen in people with a higher CD4 count (aOR: 0.27, 95% CI: 0.19–0.37, 200–499 vs. <200, *P* < 0.001; aOR: 0.26, 95% CI: 0.15–0.42, ≥500 vs. <200, *P* < 0.001) and in those taking an integrase inhibitor-based regimen (aOR: 0.55, 95% CI: 0.35–0.83, *P* = 0.007; vs those taking an NNRTI-based regimen) after adjustment. Higher odds of virologic failure were revealed among males (aOR: 2.32; 95% CI: 1.11–5.67; *P* = 0.039; male vs. female).

**Table 4:** Logistic regression for virologic failure.

Characteristics	Univariate		Multivariate	
	OR (95% CI)	*P*	aOR (95% CI)	*P*
ART initiation group				
≥30 days	1		1	
8–29 days	0.81 (0.6, 1.1)	0.175	0.76 (0.56, 1.04)	0.089
≤7 days	0.5 (0.28, 0.92)	0.027	0.50 (0.26, 0.89)	0.028
Sex				
Female	1		1	
Male	1.96 (0.92, 4.2)	0.083	2.32 (1.11, 5.67)	0.039
Age at enrollment (years)				
18–29	1		1	
30–49	1.55 (0.98, 2.45)	0.060	1.06 (0.65, 1.78)	0.825
≥50	1.97 (1.21, 3.2)	0.006	1.10 (0.60, 2.05)	0.769
Enrollment CD4, (cells/μL)				
<200	1		1	
200–499	0.27 (0.2, 0.38)	<0.001	0.27 (0.19, 0.37)	<0.001
≥500	0.26 (0.15, 0.43)	<0.001	0.26 (0.15, 0.42)	<0.001
Transmission route				
Homosexual	1		1	
Heterosexual	1.12 (0.81, 1.53)	0.501	1.09 (0.77, 1.52)	0.639
Injection drug use	1.91 (0.59, 6.19)	0.278	1.49 (0.35, 4.26)	0.518
Unknown	0.7 (0.22, 2.24)	0.553	0.56 (0.14, 1.54)	0.336
Marital status				
Single	1		1	
Married/cohabiting	1.51 (1.1, 2.09)	0.012	1.26 (0.83, 1.91)	0.271
Divorced/widowed	1.57 (1.03, 2.37)	0.034	1.26 (0.77, 2.02)	0.347
Initial ART regimen				
NNRTI-based	1		1	
PI-based	0.42 (0.1, 1.71)	0.226	0.32 (0.05, 1.03)	0.116
INSTI-based	0.62 (0.4, 0.94)	0.025	0.55 (0.35, 0.83)	0.007

*ART*, antiretroviral therapy; *OR*, odds ratio; *CI*, confidence intervals; *aOR*, adjusted odds ratio; *IQR*, interquartile ranges; *NNRTI*, non-nucleoside reverse transcriptase inhibitor; *PI*, protease inhibitor; *INSTI*, integrase inhibitor.

## Discussion

We investigated the relationship between rapid ART initiation and therapeutic outcomes in China from 2016 to 2022 in this retrospective large-scale real-life study. Real-world data on rapid ART initiation are crucial, particularly for validating its benefits and hazards in routine use outside the scope of randomized controlled studies. We noticed a considerable increase in the percent of PWH starting ART rapidly after the treat-all policy was implemented nationally in China, with the percent of rapid ART individuals increasing from 4.2% in 2016 to 19.7% in 2022. Our study revealed that the rapid ART was associated with a comparable risk of LTFU but lower rates of mortality and virologic failure compared with delayed ART initiation.

After adopting the treat-all policy, 90% of individuals within our cohort initiated ART >7 days postdiagnosis, whereas 40% began ART after >30 days. Considerable obstacles persist, especially poor linkage to care, despite China’s considerable efforts to improve accessibility to ART via the incremental extension of NFATP (Cao et al., 2020). HIV monitoring and diagnosis have traditionally been handled by the China CDC, while ART and follow-up are provided by healthcare facilities (Cao et al., 2020). However, this has caused a physical barrier between the locations where patients receive long-term care and therapy and are diagnosed, creating a structural obstacle to care management. Implementing an integrated care cascade was recommended, which involves integrating diagnostic, therapies, and management services inside HIV care centers for complete care (Aids et al., 2018). However, the local implementation of the integrated care cascade in Xi’an indicates that the association of care requires further improvement. Moreover, considerable regional regarding the HIV pandemic, legislation, economic growth, and healthcare services are observed in China, potentially resulting in an extended delay in rapid ART policy adoption and full implementation.

Unlike the results of two meta-analyses (Mateo-Urdiales et al., 2019; Bai et al., 2022)—which reported that rapid ART initiators demonstrated a decreased risk of LTFU—no significant differences in LTFU were found between rapid initiation and delayed ART at 12 months after enrollment in our study. Additionally, numerous observational studies have revealed that rapid ART is related to poorer rates of engagement in care (Joseph Davey et al., 2020; Lilian et al., 2020; Puttkammer et al., 2020; Onoya et al., 2021; Kimanga et al., 2022), potentially attributed to patient- and health provider-level factors affecting ART care retention, including income level, perceived health provider expertise, varying care levels and counseling at facilities, or ancillary service accessibility. According to our findings, enhanced, individualized counseling is necessary to increase care retention among PWH initiating ART.

Our results—contradicting the results of previous studies (Murenzi et al., 2023; Ross et al., 2023)—revealed that rapid ART initiators had lower rates of virologic failure. Many studies revealed no differences in viral suppression rates between rapid and delayed ART initiation; thus, future viral suppression among PWH may not be negatively affected by the timing of ART. Even though WHO advises urging surveillance at 6 months, most Chinese PWH had no viral load data 6 months after treatment, indicating gaps in viral load monitoring (Cao et al., 2020; Organization, W.H, 2021). Further enlargement of coverage and a substantial enhancement in the capacity for viral load measurement will be necessary. The impact of rapid ART on long-term viral suppression remains uncertain. Accelerating the initiation of ART may cause early viral suppression in the long term due to enhanced ART uptake, compliance, and retention in therapy. Moreover, our study revealed that rapid ART was associated with decreased mortality compared with delayed initiation. Our results confirm that rapid ART initiation is beneficial to Chinese PWH in practical situations.

Our study has several limitations. First, it did not measure specific determinants that affect care retention. These determinants included socioeconomic status, transportation accessibility, travel distance to the hospital, knowledge regarding treatment, and wait times at the hospital. Further, this study did not evaluate tuberculosis or cryptococcal meningitis infection, for which ART ought to be postponed (Committee, W.G.R, 2017). Furthermore, the emergence of the coronavirus disease 2019 epidemic may affect the compliance of individuals with HIV receiving ART from 2020 to 2022. Finally, the study consisted of an urban cohort in China characterized by a comparatively low HIV incidence. Hence, the generalizability of our findings to other situations may be limited.

In conclusion, our findings reveal that rapid ART initiation is available in Chinese healthcare facilities, with a notable rise in the percent of PWH in Xi’an who have access to rapid ART from 2016 to 2022 after adopting the treat-all policy. Compared with delayed initiation, rapid ART initiation caused similar LTFU but lower mortality and virologic failure. This large-scale study provided real-world feasibility and efficacy data for implementing rapid ART initiation procedures in China.

## Data Availability

The original contributions presented in the study are included in the article/supplementary material. Further inquiries can be directed to the corresponding author.
